# Follow-up of GSTM1, GSTT1, and NAT2 genotyped patients with knee or hip replacement

**DOI:** 10.17179/excli2025-8565

**Published:** 2025-06-18

**Authors:** Selahattin Bozkurt, Silvia Selinski, Meinolf Blaszkewicz, Jörg Reinders, Jan G. Hengstler, Lukas Niggemann, Klaus Golka

**Affiliations:** 1 Catholic Clinics in the Märkischer Kreis, location St. Vincenz Krankenhaus, Menden, Germany; 2 Leibniz Research Centre for Working Environment and Human Factors at TU Dortmund (IfADo), Dortmund, Germany; 3 Catholic Clinics in the Märkischer Kreis, location St. Elisabeth Hospital, Iserlohn, Germany

**Keywords:** osteoarthritis, hip arthroplasty, knee arthroplasty, glutathione S-transferase M1 (GSTM1), glutathione S-transferase T1 (GSTT1), N-acetyltransferase 2 (NAT2)

## Abstract

A total of 147 patients, genotyped for glutathione S-transferases M1 (GSTM1) and T1 (GSTT1) and for N-acetyltransferase 2 (NAT2), who had undergone total joint replacement of the knee or hip joint between August 2004 and June 2007, showed with 45% a remarkably lower portion of the *GSTM1*-negative genotype compared to both a local control (51%), an external control (52%) and the portion reported in the literature for the European population (50%). In contrast, the portions of *GSTT1*-positive (84%) and slow *NAT2* (55.1%) patients of the initial collective were unremarkable, compared to both controls. To elucidate a possible impact of this interesting finding on the long-term outcome, the patients were contacted in December 2015. Afterwards, they were interviewed using a self-prepared questionnaire. The average follow-up time was 9 years. At the time of follow-up, 57 patients were deceased, 46 patients did not respond and 12 patients declined the interview. A total of 32 patients participated in the follow-up. The mean age of the followed-up patients was 75.9±8.3 years, whereas the mean age of all patients at the time of surgery was 70.9±9 years. The portions of the *GSTM1*-negative genotype (15 out of 32; 47%), the *GSTT1*-positive genotype (24 out of 32; 75%) and the slow *NAT2* status (17 out of 32; 53%) in the followed-up patients were comparable to those of the initial collective. The follow-up results of the patients after 9 years were unable to clarify the significance of the observed lower portion of *GSTM1*-negative patients. In view of a recently published omics study reporting a reduced GSTM1 activity in tissue attached on hip implants explanted due to aseptic loosening, the striking portion of the *GSTM1*-negative genotype in this present study may encourage further investigation into the impact of this gene in patients with hip or knee replacement.

## Introduction

Osteoarthritis of the knee or hip joints is a common condition in the general population and is becoming increasingly prevalent as the population ages. It is also of considerable socio-medical importance due to the high costs of both treatment and sick leave. In 2022, 177,826 initial hip joint implants and 137,030 initial knee joint implants were documented in Germany. In addition, there were 18,145 follow-up hip joint procedures and 14,379 follow-up knee joint procedures (Endoprothesenregister Deutschland (EPRD), 2023[11]).

Conservative treatment of osteoarthritis cannot currently reverse cartilage damage, as joint cartilage does not regenerate. This is impressively demonstrated by a study on the uptake of the radioactive carbon isotope ^14^C, which originated from surface nuclear weapons tests and, unlike most other tissues, was not increasingly incorporated into joint cartilage (Libby et al., 1964[40]). Therefore, in addition to taking pressure off the arthritic joint, conservative treatment primarily aims to relieve the pain.

When pain therapy has reached its limits, the only remaining effective therapeutic option is joint replacement. As the service life of artificial joints is limited (Evans et al., 2019[13][12]), it is of considerable interest to investigate factors that could be associated with a non-surgically induced reduced service life of the implants.

Aseptic loosening is the most common cause of total hip replacement and total knee replacement failure and revision surgery (Koks et al., 2020[34]). However, there is still no effective therapeutic target regarding prevention or conservative treatment. Although the impact of genetic factors on the development of osteoarthritis in the hip or knee joint is well known from twin studies (MacGregor et al., 2000[45]; Page et al., 2003[54]; Möller et al., 2015[47]; Skousgaard et al., 2016[62]), the underlying mechanism of aseptic loosening is largely unknown (Deng et al., 2017[7]). In contrast, Pan et al. (2014[55]) stated that genetic factors play an important role in early failure of total hip replacement due to aseptic loosening, e.g., SNPs (single-nucleotide polymorphisms) of *GNAS1* (*guanine nucleotide-binding protein, alpha-stimulating activity polypeptide 1*), *TNF*-238 (*tumor necrosis factor-alpha (TNF-α) *238 G/A promoter polymorphism), *TNF-α* (*tumor necrosis factor-alpha*), *IL-6-*174 (*interleukin-6* 174 G/C promoter polymorphism), *MMP1* (*matrix metalloproteinase-1*), and *MMP2* (*matrix metalloproteinase-2*).

However, several results of the first studies, e.g., the impact of *GNAS1* or *TNF* genes on aseptic loosening could not be confirmed by later analyses (Koks et al., 2020[34]). A break-through in this area was a GWAS (genome-wide association study) based on 423 patients with total joint replacement, who were divided into 3 groups (without symptoms who received an implant at least one year before, patients undergoing implant surgery, patients receiving revision due to aseptic loosening) (Koks et al., 2020[34]). Totally 52 SNPs with a genome-wide suggestive p value less than 10^-5^ were associated with implant loosening. The highest odds ratios (OR) were found for variants in the *IFIT2*/*IFIT3 *(*interferon-induced protein with tetratricopeptide repeats 2* and *3*, resp.) (OR 21.6, p= 4.35x10^-5^), *CERK* (*ceramide kinase*) (OR 12.6, p=7.51x10^-7^), and *PAPPA* (*pappalysin*) (OR 14.0, p=1.27x10^-5^) genes. Variants of the 4 SNPs rs115871127, rs16823835, rs13275667 and rs2514486 predicted variability in the time to aseptic loosening.

In this study we focused on the genotype frequencies of the polymorphic xenobiotic-metabolising enzymes GSTM1 (glutathione S-transferase M1), GSTT1 (glutathione S-transferase T1), and NAT2 (N-acetyltransferase 2) in the follow-up group of patients with knee or hip replacement.

**Figure 1:**
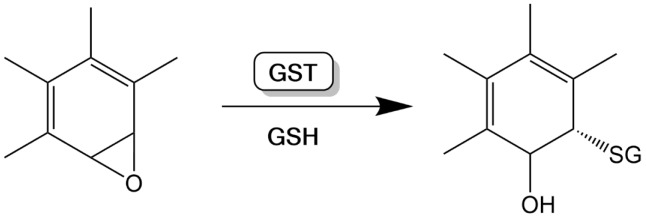
Detoxification of an epoxide by glutathione S-transferase (GST) catalysed GSH conjugation (based on Deutsche Forschungsgemeinschaft, 2008)

The gene coding for glutathione S-transferase M1 (GSTM1) is located on chromosome 1 and has a length of 21,226 bases (Gene Cards The human genome database, 2025[16]). The frequency of the GSTM1-negative genotype in Europeans is 50%. It ranges from 27% (Sub-Saharan Africans) to 54% (East Asians) (Nakanishi et al., 2022[48]). The enzyme GSTM1 metabolises highly reactive molecules (Bolt and Thier, 2006[5]; Ginsberg et al., 2009[20]). A classic example is epoxides resulting from polycyclic aromatic hydrocarbons metabolism by CYP450 (cytochrome P450) enzymes such as CYP1A1/2 or CYP1B1 (Figure 1(Fig. 1); Reference in Figure 1: Deutsche Forschungsgemeinschaft, 2008[8]). 

A special feature of the enzyme GSTM1 is that it is obviously only relevant for the detoxification of substances whether the gene is present or not. However, single-nucleotide exchanges were also detected in this gene (Gene Cards The human genome database, 2025[16]). The first report on bladder cancer and *GSTM1* showed an association with smoking habits (Bell et al., 1993[2]). Later, an association with environmental and / or occupational exposure was reported (Golka et al., 1997[22], 2009[21]; Hung et al., 2004[28]; Figueroa et al., 2015[14]). This association was no longer detectable after the end of exposure in the regions of Dortmund, Germany and Lutherstadt Wittenberg, Germany due to industrial structural changes with a dramatic reduction in emissions of combustion products due to reduction in the enzyme`s substrate (Ovsiannikov et al., 2012[53]; Krech et al., 2017[36]; Bergmann et al., 2025[3]). In a recent meta analysis based on 28,270 bladder cancer cases from 46 studies, OR was 1.37 (95% CI 1.19-1.59) for smokers and 1.26 (95% CI 1.08-1.48) for non-smokers (Yu et al., 2017[66]).

However, to date there have been hardly any studies on the role of this enzyme in osteoarthritis. Klein et al. (2012[33]) found a remarkably reduced portion of *GSTM1*-negative patients (45%) in 147 patients with hip or knee joint replacement compared to two control groups (local 51%, external 52%) and compared to the portion reported in the literature for the European population (50%) (Nakanishi et al., 2022[48]). In addition, the association of the *GSTM1* genotype with cytogenetic abnormalities was analysed in knee joint synovial cells from patients with arthritis of different etiology (Ilyinskikh et al., 2017[29]) and in joint synovial fluid with the recovery of patients undergoing hip replacement (Lin et al., 2018[41]). GSTM1 also deactivates reactive oxygen species and lipid peroxidation products (Ellwanger et al., 2025[10]). For a comprehensive overview of the impact of the *GSTM1* gene on different diseases, see Nakanishi et al. (2022[48]).

The gene coding for glutathione S-transferase T1 (GSTT1) is located on chromosome 22 and has a length of 8,179 bases (Gene Cards The human genome database, 2025[17]). The frequency of *GSTT1*-negative genotype in Europeans is 17%. It ranges from 14 % (Native Americans) to 48 % (East Asians) (Nakanishi et al., 2022[48]). An essential task of this enzyme is the conjugation of reduced glutathione to a wide number of exogenous and endogenous hydrophobic electrophiles (Gene Cards The human genome database, 2025[17]).

GSTT1 metabolises in particular small molecules with one or two carbon atoms, such as dichloromethane or ethylene oxide (Bolt and Thier, 2006[5]). A special feature of GSTT1 is that, as with the above described enzyme GSTM1, it is obviously only relevant for the detoxification of substances whether this gene is present or not. GSTT1 also deactivates reactive oxygen species and lipid peroxidation products (Ellwanger et al., 2025[10]). For a comprehensive overview of the impact of the *GSTT1* gene on different diseases, see Nakanishi et al. (2022[48]).

The gene coding for N-acetyltransferase 2 (NAT2) is located on chromosome 8 and has a length of 14,918 bases (Gene Cards The human genome database, 2025[18]). Over 120 alleles (haplotypes) are known to date (NAT Committee, 2024[49]). With regard to the effect in the organism, a distinction is generally made between rapid and slow acetylation. Ruiz et al. (2012[57]) described an ultra-slow NAT2 status for the first time. Selinski et al. (2013[60]) reported that the ultra-slow NAT2 status increases the risk of urinary bladder cancer even if the classic slow NAT2 acetylation status, which includes all genotypes coding for a slow NAT2 acetylation status, no longer shows an increased risk of urinary bladder cancer. Compared to the slow NAT2 acetylation status, the ultra-slow NAT2 acetylation status has a metabolic capacity that is reduced by approx. 30% (Selinski et al., 2013[60]). This means that in ultra-slow NAT2 acetylators, the oxidative metabolic pathway is followed to a relevant extent, which ultimately leads to arylnitrenium ions, which, according to current knowledge, are the metabolites of carcinogenic aromatic amines that trigger bladder cancer (Hein et al., 1992[26]). 

Classical substrates of NAT2 are aromatic amines and other substances that have an amino group on an aromatic ring or in which such a group is released during metabolism (Golka et al., 2004[24]). The substrates of NAT2 also include, for example, the tuberculostatic drug isoniazid (INH), the anticonvulsant clonazepam, the antihypertensive drug hydralazine and the anti-inflammatory drug sulfasalazine (Gene Cards The human genome database 2025[18]).

With regard to inflammatory diseases, there is a known correlation between NAT2 acetylation status and the risk of developing lupus erythematosus, an autoimmune inflammatory rheumatic disease from the group of collagenoses (von Schmiedeberg et al., 1999[65]; Santos et al., 2016[58]). In psoriasis, which is also an autoimmune disease and manifests as an inflammatory skin disease, an association between two SNPs of *NAT2* and an earlier manifestation as well as a more severe course has been described (Dursun et al., 2018[9]). To the best of our knowledge, no studies have yet been published on a possible association between osteoarthritis of the hip or knee joint and NAT2 acetylation status.

The aim of this study was to investigate the frequencies of *GSTM1*, *GSTT1*, and *NAT2* genotypes in the follow-up group.

## Materials and Methods

This follow-up study is based on a study group of 147 patients who had undergone total joint replacement of the knee or hip joint between August 2004 and June 2007 at the department of surgery, St. Elisabeth Hospital, Iserlohn, Germany (Klein et al., 2012[33]). First, the patients were contacted in December 2015 and then interviewed using a self-prepared questionnaire, given in the supplement. The control group of 129 patients without indication for joint replacement of the initial study group was from the surgical department of the St. Elisabeth Hospital, Iserlohn, Germany (Klein et al., 2012[33]). *NAT2* genotypes of the Iserlohn patients were separately published (Selinski et al., 2011[61]). The external control group of urological patients without known cancer was from the department of urology of the Josefs Hospital, Dortmund, Germany (Ovsiannikov et al., 2012[53]), which is about 25 km away.

The study was approved by the Ethics Committee of the Westphalia-Lippe Medical Association and of the Westphalian Wilhelms University, Münster (2015-492-f-S) and by the Institutional Review Board.

### Analytical Methods 

Genotyping for *GSTM1*, *GSTT1* and *NAT2* was performed during the initial study period between August 2004 and June 2007 according to standard methods. In short, DNA of cases and controls was isolated from venous blood samples according to standard methods in the Dortmund Institute (QIAamp DNA Maxi Kit Qiagen, Hilden, Germany). Genotyping for the presence or absence of the *GSTM1* and GSTT1 genes was performed by duplex polymerase chain reaction (PCR), as described by Kempkes et al. (1996[31]). *NAT2* genotyping was performed by PCR and RFLP-based methods (Selinski et al., 2013[60]).

### Statistical Methods

Percentages of the different genotypes of the three investigated polymorphic enzymes GSTM1, GSTT1 and NAT2 were determined in the groups of followed-up, deceased and not contactable patients. Age at death of the deceased patients with artificial knee or hip replacement was plotted using Kaplan-Meier curves. The free software R version 4.2.1 was used for all calculations and plotting Kaplan-Meier curves (The R Foundation, 2022[63]).

## Results

**Figure 2:**
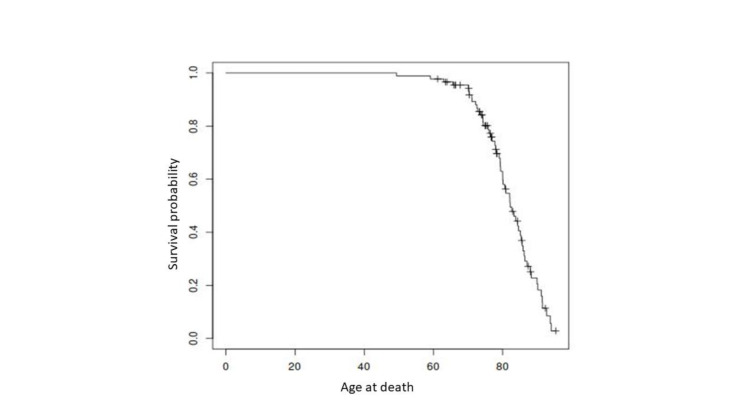
Age at death of the 57 deceased patients with artificial knee or hip replacement

At the time of follow-up, 57 patients were deceased, 46 patients did not respond and 12 patients declined the interview. The age of the deceased patients is presented in Figure 2(Fig. 2). A total of 32 patients participated in the follow-up. The average follow-up time was 9 years.

The mean age of the followed-up patients was 75.9±8.3 years, whereas the mean age of all patients at the time of surgery was 70.9±9 years. 

**Figure 3:**
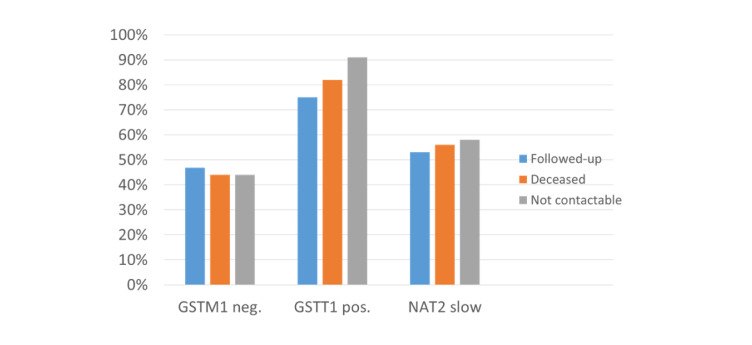
Portions of *GSTM1*-negative, *GSTT1*-positive, and *slow *NAT2 genotypes in followed-up, deceased, and not contactable patients with artificial knee or hip replacement

**Figure 4:**
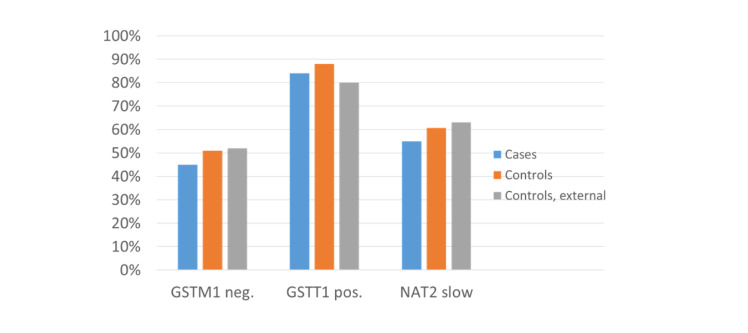
Portions of *GSTM1*-negative, *GSTT1*-positive, and slow *NAT2* genotypes in the initial collective with artificial hip or knee replacement, in local hospital and in external controls (Klein et al., 2012; Ovsiannikov et al., 2012; Selinski et al., 2011)

The portions of the *GSTM1*-negative genotype (15 out of 32; 47%), the *GSTT1*-positive genotype (24 out of 32; 75%) and the slow *NAT2* status (17 out of 32; 53%) in the followed-up patients (Figure 3(Fig. 3)) were comparable to that of the initial collective that showed with 45% a remarkably lower portion of the *GSTM1*-negative genotype compared to both a local control (51%) and an external control (52%) (Figure 4(Fig. 4); References in Figure 4: Klein et al., 2012[33]; Ovsiannikov et al., 2012[53]; Selinski et al., 2011[61]) and the portion reported in the literature for the European population (50%) (Nakanishi et al., 2022[48]). The portions of *GSTT1*-positive (84%) and slow *NAT2* (55.1%) patients of the initial collective were unremarkable, compared to both controls.

A review of the routine postoperative X-ray images of the joints revealed that although they provided the information required by the surgeon to assess the correct position of the implant, they did not provide any relevant additional information for the study.

## Discussion

Twin studies showed the impact of genetic factors on the development of osteoarthritis in the hip or knee joint (MacGregor et al., 2000[45]; Page et al., 2003[54]; Möller et al., 2015[47]; Skousgaard et al., 2016[62]). However, the genetic effect was reported to be greater in the case of osteoarthritis of the hip joint (Magnusson et al., 2017[46]). Besides a considerable number of articles, several GWAS have been published on the impact of polymorphisms (Kerkhof et al., 2010[32]; arcOGEN Consortium, 2012[1]; Boer et al., 2021[4]; Kulm et al., 2022[38], 2023[37]; Henkel et al., 2023[27]; Hatzikotoulas et al., 2025[25]). 

Henkel et al. (2023[27]) described 28 variants for knee joint osteoarthritis and 34 variants for hip joint osteoarthritis in a GWAS based on 61,151 cases of knee joint osteoarthritis, including 22,522 cases with joint replacement, and 38,068 cases of hip joint osteoarthritis, including 20,221 cases with joint replacement. Among them, no genes coding for xenobiotic-metabolising enzymes were reported.

The observed odds ratios were low. For surgically treated knee joints, the odds ratios for the variants in the additive model were a maximum of 1.11; for the only variant analysed according to the recessive model, the odds ratio was 3.57. For surgically treated hip joint arthrosis, the odds ratios were significantly higher. Here, in the analysis according to the recessive model, 6 variants were above 1.11, with a maximum of 2.56. In the three variants analysed according to the recessive model, the odds ratio was between 1.14 and 5.60.

Most recently, Hatzikotoulas et al. (2025[25]) published a GWAS based on 489,975 osteoarthritis cases, including all locations of osteoarthritis, and 1,472,094 controls. This study included 97,328 hip osteoarthritis cases, 49,874 total hip replacement cases, 172,256 knee osteoarthritis cases, and 48,161 total knee replacement cases. The authors reported an association of 146 variants for knee joint osteoarthritis, 92 variants for total knee replacement, 151 variants for hip joint osteoarthritis and 136 variants for total hip replacement. Furthermore, they identified eight biological processes enriched for effector genes. Although the study of Hatzikotoulas et al. (2025[25]) included drug targets, only *CYP26B1* (*cytochrome P450 26B1*) involved in the degradation and *ALDH1A2* (*aldehyde dehydrogenase 1A2*) involved in the synthesis of all-*trans*-retinoic acid were reported as genes coding for metabolizing enzymes associated with hip or knee osteoarthritis. Furthermore, a decrease in *COX1* *(cytochrome C oxidase 1*, synonym: *cyclooxygenase-1*) (synonym*: PTGS1*, *prostaglandin-endoperoxide synthase 1*) expression in degraded compared with intact osteoarthritis-affected chondrocytes was reported.

To overcome the generally low power of single-nucleotide polymorphisms (Golka et al., 2011[23]), polygenic risk scores (PRS) have been applied (Sedaghati-Khayat et al., 2022[59]). On the other hand, Gill et al. (2024[19]) reported that reduced genomic heterozygosity is associated with a higher risk of osteoarthritis. Hatzikotoulas et al. (2025[25]) also evaluated the predictive potential of risk scores. The best performing genetic risk score (GRS) was obtained for hip osteoarthritis (area under the curve, AUC, 58.6%). Therefore, such scores will not achieve a noticeable practical significance beyond research.

Studies on the impact of polymorphisms on the service life of the implant and/or long-term satisfaction with the implant have not been published, with the exception of studies of Omar et al. (2017[52]), who applied transcriptome-wide high-density microarray analysis on periprosthetic tissue from 12 hips with chronic infection, using 12 hips with aseptic loosening as controls, and Liu et al. (2025[42]). Liu et al. (2025[42]) applied integrated transcriptomics, proteomics and untargeted metabolomics analyses in 8 cases of aseptic loosening of implants after hip replacement. They observed characteristic metabolite changes (biosynthesis of guanine, L‑glycine and adenosine) and decreased CRLF1 (cytokine receptor-like factor 1) and GSTM1 activities in tissues attached to explanted artificial hip specimens, compared to 8 controls who received primary total hip replacement due to avascular necrosis of femoral head or femoral neck fracture. 

Service life of the implant and satisfaction with an artificial knee or hip joint can vary greatly. Early loosening of implants is essentially caused by five interfering factors: patient, material, implant design, fixation, and surgical technique (Katzer and Löhr, 2003[30]). However, the underlying mechanism of aseptic loosening is largely unknown (Deng et al., 2017[7]). Polymorphic enzymes do not only influence a number of diseases with regard to the risk of disease (NHGRI-EBI GWAS Catalog, 2025[50]), but in some cases also its course. 

In the initial study, patients were genotyped for glutathione S-transferases M1 (GSTM1) and T1 (GSTT1) as well as for N-acetyltransferase 2 (NAT2) to investigate the impact of the polymorphic enzymes on the indication for joint replacement (Klein et al., 2012[33]) - pain that can no longer be controlled with medication. These patients showed a remarkably lower portion of the *GSTM1*-negative genotype (45%) compared to two control groups (local 51%, external 52%) and the portion reported in the literature for the European population (50%) (Nakanishi et al., 2022[48]).

In the scientific discussion with colleagues, the question raised was what the lower frequency of the *GSTM1*-negative genotype and thus an increased metabolic activity with regard to the detoxification of reactive metabolic products could mean in the long term with regard to the service life of the joint replacement and patient satisfaction. 

Apart from the study by Klein et al. (2012[33]), no studies listed in PubMed or Google Scholar on the frequency of the *GSTM1*-negative genotype in this patient group have been published to date. 

One reason for this could be that this genotype was not included as standard on SNP chips used in GWAS, at least not in the past. This is because a decisive criterion for the inclusion of a reference SNP or relevant genetic information on a SNP chip is the good differentiation of the signal. As an example, this meant that previously the ultra-slow *NAT2* genotype defined by SNPs rs1041983 and rs1799930 was not genotyped in GWAS for bladder cancer (Figueroa et al., 2016[15]) - even though the slow NAT2 phenotype is the oldest known genetic risk factor for the development of urinary bladder cancer when exposed to carcinogenic aromatic amines (Lower et al., 1979[43]). Due to its fundamental meaning in molecular epidemiology, the article of Lower et al. (1979[43]) was reprinted and commented (Lower et al., 2007[44]; Vineis, 2007[64]; Olden, 2007[51]; Rothman et al., 2007[56]).

The *GSTM1* genotype, which is a risk factor for bladder cancer, was therefore determined indirectly via a proxy marker in some GWAS for bladder cancer, for example. A proxy marker (chr1:110229772) effectively tagged the *GSTM1* deletion (Koutros et al., 2023[35]).

In a patient population with an average age of 70.9±9 years, the question arises as to when a follow-up should be carried out. A higher probability of participation speaks in favour of a short follow-up period, where lower number of deaths in the patient population as well as the robustness of the contacted patients is to be expected. An argument against a relatively short follow-up period is that no findings can be obtained on the long-term stability of the implants and the long-term satisfaction of the patients with regard to quality of life. 

In the present study, the follow-up period was 9 years. During this period, 57 patients had died and 46 could not be contacted.

The selected follow-up period covers at least a considerable part of the period in which early implant loosening occurs. The frequency of failure of hip replacements varies among countries. During the first 5 years, New Zealand had the highest survival rate, with almost 97%, while Denmark had the lowest with 95.5%. After 10 years, Australia revealed the highest survival rate of implants (95%), whereas Denmark had the lowest, namely 92.8%. Fifteen years after implantation, 94% of hip replacements in Australia survived, whereas the survival rate in Denmark was much lower with 89% (Clar et al., 2024[6]). These findings are also of clinical interest in patient collectives at an older age. However, early loosening is not clearly defined (Kvarda et al., 2024[39]).

The main result of this study is that the portion of *GSTM1*-negative patients (47%) is comparable to the portion in the initial study group, albeit with a small number of cases. The conspicuous distribution in the follow-up group does not provide any concrete evidence of a possible cause.

The reduced GSTM1 activity reported by Liu et al. (2025[42]) in cells attached to explanted artificial joints due to early loosening may also result from non-genetic causes such as implant-related changes in the microenvironment. Therefore, factors like substances released from the artificial joint or the cement used or biological processes triggered by the implants may be considered. 

Overall, with regard to the possible systemic influence of *GSTM1* on osteoarthritis of the knee or hip joint, it must also be taken into account that noticeable associations with SNPs may be caused by indirect effects, such as an impact on obesity risk factors.

In view of the first description of a decreased activity of the GSTM1 enzyme in tissues attached to explanted artificial hip specimens from patients with aseptic loosening of the artificial hip joint compared to controls by Liu et al. (2025[42]), the findings of this study and the preceding study by Klein et al. (2012[33]) may encourage further research on the impact of *GSTM1* in patients with hip or knee replacement.

## Notes

*Dedicated to our librarian Dipl.-Bibl. Susanne Lindemann on the occasion of her retirement*

## Declaration

### Author Contributions

Conceptualization S.B., S.S., J.G.H., and K.G., methodology S.S., M.B., and J.R., software S.S., laboratory analysis M.B., data analysis S.B., S.S., investigation S.B., S.S., and K.G., resources L.N., J.G.H., data curation J.R., S.S., writing-original draft preparation S.B., S.S., M.B., and K.G., writing-review and editing S.B., L.N., and K.G., visualization S.B., K.G., supervision L.N., J.G.H, project administration J.G.H., K.G. All authors have read and agreed to the published version of the manuscript. 

### Funding

This research received no external funding. 

### Institutional Review Board Statement

The study was conducted in accordance with the Declaration of Helsinki, and approved by the Institutional Review Board and by the Ethics Committee of the Westphalia-Lippe Medical Association and of the Westphalian Wilhelms University, Münster (2015-492-f-S).

### Informed Consent Statement

Informed consent was obtained from all subjects involved in the study.

### Data Availability Statement

The data are available on reasonable request from the corresponding author.

### Conflicts of Interest

The authors declare no conflict of interest.

### Using Artificial Intelligence (AI)

The authors have not used artificial intelligence-assisted technologies.

## Supplementary Material

Supplementary data
