# Supplementary material for: Follow-up of GSTM1, GSTT1, and NAT2 genotyped patients with knee or hip replacement
Source: EXCLI J. 2025 Jun 18;24:677–89. doi: 10.17179/excli2025-8565 (PMC12235267; doi:10.17179/excli2025-8565)
Supplement: Supplementary data [file EXCLI-24-677-s-001.pdf]

EXCLI Journal 2025;24:677-689 – ISSN 1611-2156
Received: May 20, 2025, accepted: June 06, 2025, published: June 18, 2025

Supplementary data to:

Original article:

FOLLOW-UP OF GSTM1, GSTT1, AND NAT2 GENOTYPED PATIENTS WITH KNEE OR HIP REPLACEMENT[#]

Selahattin Bozkurt[1], Silvia Selinski[2], Meinolf Blaszkewicz[2], Jörg Reinders[2], Jan G. Hengstler [2], Lukas Niggemann[3], Klaus Golka[2*]

[1] Catholic Clinics in the Märkischer Kreis, location St. Vincenz Krankenhaus, Menden, Germany; se.se.boz06@gmail.com
[2] Leibniz Research Centre for Working Environment and Human Factors at TU Dortmund (IfADo), Dortmund, Germany; s.selinski@gmx.de; blaszkewicz@t-online.de; reinders@ifado.de; hengstler@ifado.de; golka@ifado.de
[3] Catholic Clinics in the Märkischer Kreis, location St. Elisabeth Hospital, Iserlohn, Germany; l.niggemann@kkimk.de

* Correspondence: golka@ifado.de Tel: ++49 (0)231 413 411

[#] *Dedicated to our librarian Dipl.-Bibl. Susanne Lindemann on the occasion of her retirement*

https://dx.doi.org/10.17179/excli2025-8565

This is an Open Access article distributed under the terms of the Creative Commons Attribution License (https://creativecommons.org/licenses/by/4.0/).

EXCLI Journal 2025;24:677-689 – ISSN 1611-2156
Received: May 20, 2025, accepted: June 06, 2025, published: June 18, 2025

Follow-up of patients with knee or hip replacement

Doctor's section

Personal statistics:

1. Gender

☐ female

☐ male

2. Year of birth

19☐☐

3. Height (in cm)

☐☐☐ cm

4. Weight (in kg)

☐☐☐ kg

5. BMI

☐☐

Details of joint replacement

6. Which joint was operated on and when?

Hip joint in 200☐ ☐ right ☐ left

EXCLI Journal 2025;24:677-689 – ISSN 1611-2156
Received: May 20, 2025, accepted: June 06, 2025, published: June 18, 2025

Knee joint in 200☐ ☐ right ☐ left

7. Was rehabilitation recommended by the clinic after the operation?

☐ outpatient ☐ inpatient ☐ no rehabilitation

8. Have complications occurred after joint replacement?

a) Fever ☐ yes ☐ no

b) Signs of infection in the operated joint ☐ yes ☐ no

c) Dislocation/dislocation ☐ yes ☐ no

d) Surgical revision ☐ yes ☐ no

9. Preoperative X-ray findings

a) Osteoarthritis stage

b) Osteoporosis ☐ yes ☐ no

10. Postoperative X-ray findings

a) Prosthesis position

☐ cemented ☐ cementless ☐ hybrid (both)

EXCLI Journal 2025;24:677-689 – ISSN 1611-2156
Received: May 20, 2025, accepted: June 06, 2025, published: June 18, 2025

Patient section

Dear patient, in the year 200__ you had the following joint operated on or had it operated on shortly before::

Hip ☐right ☐left **Knee** ☐right ☐left

1. Was rehabilitation initiated by the clinic after the operation?

☐ outpatient ☐ inpatient ☐ no rehabilitation

2. How long did you need pain medication after the operation? Which medication was taken?

Duration of use:____

Medication:____

3. Were the pain and mobility improved compared to before the operation?

☐ no ☐ slightly ☐ moderately ☐ significantly

4. Have you noticed a deterioration of the initial result on the operated joint over the years?

☐ no ☐ slightly ☐ moderately ☐ significantly

EXCLI Journal 2025;24:677-689 – ISSN 1611-2156
Received: May 20, 2025, accepted: June 06, 2025, published: June 18, 2025

5. What statements can you make about the joint function of the above-mentioned joint?

	Before surgery	After surgery	Currently
**Initial pain:**	☐ none<br>☐ occasionally<br>☐ frequently<br>☐ constantly	☐ none<br>☐ occasionally<br>☐ frequently<br>☐ constantly	☐ none<br>☐ occasionally<br>☐ frequently<br>☐ constantly
**Pain on movement:**	☐ none<br>☐ occasionally<br>☐ frequently<br>☐ constantly	☐ none<br>☐ occasionally<br>☐ frequently<br>☐ constantly	☐ none<br>☐ occasionally<br>☐ frequently<br>☐ constantly
**Rest pain:**	☐ none<br>☐ occasionally<br>☐ frequently<br>☐ constantly	☐ none<br>☐ occasionally<br>☐ frequently<br>☐ constantly	☐ none<br>☐ occasionally<br>☐ frequently<br>☐ constantly
**Mobility:**	☐ not restricted	☐ not restricted	☐ not restricted

EXCLI Journal 2025;24:677-689 – ISSN 1611-2156
Received: May 20, 2025, accepted: June 06, 2025, published: June 18, 2025

	☐ slightly restricted<br><br>☐ moderately re-stricted<br><br>☐ severely re-stricted	☐ slightly re-stricted<br><br>☐ moderately re-stricted<br><br>☐ severely re-stricted	☐ slightly re-stricted<br><br>☐ moderately re-stricted<br><br>☐ severely re-stricted

	**Before surgery**	**After surgery**	**Currently**
**Pain medication requirements:**	☐ none<br><br>☐ occasionally<br><br>☐ frequently<br><br>☐ constantly	☐ none<br><br>☐ occasionally<br><br>☐ frequently<br><br>☐ constantly	☐ none<br><br>☐ occasionally<br><br>☐ frequently<br><br>☐ constantly
**Physical activity in everyday life:**	☐ not restricted<br><br>☐ slightly re-stricted<br><br>☐ moderately re-stricted<br><br>☐ severely re-stricted	☐ not restricted<br><br>☐ slightly re-stricted<br><br>☐ moderately re-stricted<br><br>☐ severely re-stricted	☐ not restricted<br><br>☐ slightly re-stricted<br><br>☐ moderately re-stricted<br><br>☐ severely re-stricted
**Walking distances in everyday life:**	☐ only a few m	☐ only a few m	☐ only a few m

	☐ limited to home<br>☐ up to 250 m<br>☐ more than 250 m	☐ limited to home<br>☐ up to 250 m<br>☐ more than 250 m	☐ limited to home<br>☐ up to 250 m<br>☐ more than 250 m

6. Have complications occurred after joint replacement?

a) Dislocation / luxation: ☐ yes ☐ no

b) Re-operation of the joint: ☐ yes ☐ no

c) Broken bone in the operated leg: ☐ yes ☐ no

d) Prosthesis loosening: ☐ yes ☐ no

7. Do you suffer from bone loss (osteoporosis)?

☐ yes ☐ no ☐ not known

8. Result of the last check-up?

Prosthesis: ☐ inconspicuous ☐ loosened ☐ other

Year of last check-up:

9. Would you undergo the operation again?

☐ definitely ☐ maybe ☐ never ☐ already done

EXCLI Journal 2025;24:677-689 – ISSN 1611-2156
Received: May 20, 2025, accepted: June 06, 2025, published: June 18, 2025

10. How satisfied are you with the result of the operation?

☐ very satisfied ☐ satisfied ☐ dissatisfied

11. Did the joint replacement at the time improve your quality of life?

☐ yes, but only in the short term ☐ yes, permanently ☐ no

12. Comment / suggestion based on your own experience

....

....

....

....

....

....

....

Thank you very much!
